# Editorial: New molecular pathways in thyroid cancer and pathophysiology: role of coding and noncoding genes

**DOI:** 10.3389/fendo.2024.1404305

**Published:** 2024-04-05

**Authors:** Cesar Seigi Fuziwara, Juan Pablo Nicola, Murilo Vieira Geraldo

**Affiliations:** ^1^ Department of Cell and Developmental Biology, Institute of Biomedical Sciences, University of São Paulo, São Paulo, Brazil; ^2^ Department of Clinical Biochemistry, Faculty of Chemical Sciences, National University of Córdoba, Córdoba, Argentina; ^3^ Clinical Biochemistry and Immunology Research Center - National Scientific and Technical Research Council (CIBICI-CONICET), Córdoba, Argentina; ^4^ Department of Structural and Functional Biology, State University of Campinas, São Paulo, Brazil

**Keywords:** thyroid cancer, coding genes, noncoding genes, molecular marker, omic analyses, mutation–genetics

Thyroid cancer is the most common endocrine malignancy arising from different cell types that compose thyroid gland, namely, follicular cells and C-cells or parafollicular cells. Within the follicular cell-derived thyroid cancer, several variants can be identified that exhibit heterogeneous behavior ranging from indolent papillary thyroid cancer (PTC) to very aggressive and lethal anaplastic thyroid cancer (ATC), turning thyroid cancer into a fruitful field for investigation of tumor biology. In this context, the Research Topic “*New molecular pathways in thyroid cancer and pathophysiology: role of coding and noncoding genes*” compiled several articles that provided novel aspects of thyroid cancer biology, adding new layers to the complexity of the disease.

Regarding the oncogenesis aspect, new molecular players have been identified using animal models, human samples and proteomic analysis. For example, Minna et al. reported mutations in *DICER1* in follicular-patterned RAS-like tumors without any oncogenic activation of the MAPK pathway. Dicer1 is an endoribonuclease that processes endogenous miRNA precursors into mature miRNA, and mutations that alter Dicer’s functionality impair this process, with consequences for cell biology. Didier-Mathon et al. reported that the *Borealin* gene (*CDCA8*), discovered in a patient with congenital hypothyroidism due to thyroid dysgenesis, is involved in thyroid cell biology. Inactivation of Borealin induces goiter and the formation of papillary-like structures that overactivate ERK signaling and induce a BRAF-like gene expression signature in transgenic mice, resembling mutation in human Borealin. Zhou et al. investigated the genetics of the non-classical PTC and revealed a high prevalence of gene fusions involving *NTRK* and *RET*, suggesting a common genetic signature among patients without BRAF or RAS mutations. Huang et al. conducted proteomic profiling of follicular-pattern thyroid tumors, and identified proteins that discriminate follicular thyroid cancer from the follicular-variant of papillary thyroid cancer. Among these proteins, ANXA1 was validated as a novel biomarker in thyroid tumors.

Currently, an array of open-access databases generated from large-scale studies are available for researchers to explore, re-analyze, and gain new insights into thyroid cancer progression. In particular, Zhang et al. used available microarray datasets to detect differentially expressed genes in metastatic PTC and identified a signature of four genes associated with iodine metabolism in metastatic PTC that were associated with poor overall survival. Liu et al. conducted bioinformatic analysis in The Cancer Genome Atlas database for thyroid cancer to identify genes associated with lymph node metastasis potential. Among a twelve-gene signature, ERBB3 (HER3) overexpression was detected in patients with lymph node metastasis or advanced stage disease, which was associated with reduced ERBB3 gene methylation.

Response to radioiodine is essential for thyroid cancer treatment and new molecular insights into the histopathology of aggressive tumors could lead to a better management in clinical practice. In this extent, Bogdanova et al. investigated aggressive radioiodine-refractory recurrent PTC and showed that while primary metastases and radioiodine-refractory metastases are less differentiated and show similar architecture with solid trabecular structure and increased p16 staining, the primary tumors are more differentiated with papillary structure. Huang et al. investigated the relationship between BRAF^V600E^ mutation and iodine avidity in distant lung metastases and showed that lymph node metastases are more likely to lose radioiodine avidity when the primary tumor harbors the BRAF^V600E^ oncogene. In addition, Mukhtar et al. investigated the association of BRAF^V600E^ and *TERT* promoter mutation in the stratification of differentiated thyroid cancer, and confirmed that only *TERT* promoter mutations, either alone or in combination with BRAF^V600E^, correlate with a high-risk disease.

To identify new vulnerabilities of aggressive thyroid cancer cells, Sriramareddy et al. and Pita et al. have investigated the efficacy of targeting DNA repair and CDK phosphorylation. Using an approach to block the DNA repair mechanism in ATC cells, Sriramareddy et al. showed that the treatment with a DNA ligase inhibitor enhanced apoptosis in doxorubicin-treated ATC cells *in vitro* and reduced tumor growth *in vivo* in nude mice. On the other hand, Pita et al. explored the potential of CDK4/6 inhibition in a comprehensive panel of thyroid cancer cell lines and observed a synergistic antitumoral effect when blocking CDK together with MAPK signaling in CDK-sensitive cells, while testing an 11-gene signature tool to detect CDK insensitivity.

Overall, we hope that these 11 articles published in the Research Topic “*New molecular pathways in thyroid cancer and pathophysiology: role of coding and noncoding genes*” have shed new light on the understanding of thyroid biology and pathogenesis, while provided new insights into this molecular field that is emerging from the interpretation of data generated in the omics era.

## Author contributions

CF: Writing – original draft, Writing – review & editing. JN: Writing – review & editing. MG: Writing – review & editing.

